# Large herbivores influence plant litter decomposition by altering soil properties and plant quality in a meadow steppe

**DOI:** 10.1038/s41598-018-26835-1

**Published:** 2018-06-14

**Authors:** Zhongnan Wang, Xia Yuan, Deli Wang, Yang Zhang, Zhiwei Zhong, Qinfeng Guo, Chao Feng

**Affiliations:** 1 Key Laboratory of Vegetation Ecology, Ministry of Education, Institute of Grassland Science/School of Environment, Northeast Normal University, Changchun, Jilin 130024 China; 2 Eastern Forest Environmental Threat Assessment Center, Southern Research Station, USDA-FS, Research Triangle Park, NC 27709 USA

## Abstract

Large herbivores act as a major driver of plant litter decomposition in grasslands. The modifications of soil biotic and abiotic properties, as well as the changes in quality (C/N ratio) of plant litter, are two key pathways by which large herbivores can affect litter decomposition. Yet we know little about the relative role of these two mechanisms in mediating decomposition. Here, by combining a large-scale and a small-scale field manipulative experiment, we examined how livestock (cattle and sheep) grazing affects standing litter decomposition of a dominant grass, *Leymus chinensis* in grasslands in northeast China. We found that livestock grazing affected litter decay rate both by its influences on soil property (soil moisture, nutrient content, and microbial communities) and on plant litter quality (C/N ratio). Due to their distinct body size and diet preference, cattle and sheep affected soil property and litter quality, thus litter decay rate, differently by causing varying disturbance regimes and by feeding on different dominant species. Our study provides evidence that herbivore grazing can influence litter decomposition by modifying soil conditions and litter quality independently. Therefore, choosing the proper large herbivore(s) in grazing regimes may be important in maintaining nutrient cycling in grassland ecosystems.

## Introduction

Most of the aboveground net primary productivity (50% to 99%) eventually enters the detrital food chain as plant litter^1,2^. Therefore litter decomposition is often regarded as a key process of nitrogen (N) and carbon (C) cycling in terrestrial ecosystems^3,4^. To date, habitat characteristics, including soil biotic and abiotic properties, and plant chemistry, particularly C/N ratio of plant tissue, have been considered as the main drivers of litter decomposition^5,6^. Large herbivores (i.e., vertebrate herbivores with body mass >30 kg^7^) are functionally important components of terrestrial ecosystems. These herbivores frequently alter vegetation biomass and composition, soil nutrients and moisture, microclimates, as well as nutrient contents in plant tissue^8–10^. Such herbivore-induced changes in habitat characteristics and plant nutrient conditions, therefore, may potentially influence decomposition of plant litter (Fig. 6)^11,12^.

Recent studies have shown that herbivore grazing can alter decomposition processes via effects on local soil conditions^13,14^. Grazing changes soil conditions (e.g. soil temperature and soil moisture) by increasing light penetration and albedo by defoliation^15^. It decreases soil aeration and water content^16,17^ but increases soil salinization^18^ by trampling. Herbivores can also directly influence soil carbon and mineral nutrients by dung and urine deposition^19,20^. These environmental effects may be partially responsible for the soil nutrients, soil physical and chemical properties, as well as the bacterial communities and their activities^21,22^. The altered conditions may subsequently impact above- and belowground decomposition rates in grazed ecosystems^23–25^. Moreover, defoliation by herbivores induces rapid changes in plant carbon and nitrogen allocation thus affecting root tissue chemistry. Defoliation can increase soil extractable carbon in the rhizosphere, which may stimulate microbial populations^26,27^. There is growing evidence that soil characteristics with different functionalities can affect litter decomposition^28,29^. However, how the soil properties modified by grazing affect the decomposition process remains unclear.

Litter quality is usually the best predictor of decomposition rates^30,31^. Litter quality includes C/N ratio and chemical composition that can be affected by large herbivores^32,33^. Most natural and managed grasslands are grazed by assemblages of different-sized herbivores with different diet preferences^34,35^. Empirical studies show that diet preference by herbivores can mediate patterns of energy and nutrient allocation at the whole-plant level^36,37^. The changes in plant nutrient and chemical composition induced by grazing may influence the quality of litter returned to soils^38–40^. Accordingly, plants with the highest nutrient availability are the main drivers of the rate of litter decomposition in an area, and these plants are also deemed to be of high palatability for herbivores^41,42^. One would expect that grazing may result in low-quality litter input into the soil thus reducing decomposition rates^43,44^. The contradicting results from previous studies suggest that herbivores can promote species that produce high-quality litter with high nutrient concentrations by means of plant regrowth following defoliation^6,45^. This process may enhance decomposition and nutrient release from litter^13,40^. Overall, such seemingly conflicting results prompt us to consider the effect of grazing on litter quality in detail.

Most current research has examined grazing effects on decomposition by changing plant species composition and climatope, but has not paid enough attention to the relative role of a single species’ litter quality vs. site conditions as a controlling factor^6,10^. In this study, we investigated how grazing-induced changes in soil characteristics and litter quality can independently influence plant standing litter decomposition of a dominant grass *L*. *chinensis* in a meadow steppe in northeast China. We also compared how different large herbivore species, namely sheep and cattle, can differently influence *L*. *chinensis* litter decomposition by changing the two pathways (soil property and litter quality) above. We address two related specific questions: (1) how litter quality and incubation site may influence litter decomposition; and (2) whether and how different herbivore species (sheep vs. cattle) may affect litter decomposition through altering litter quality and incubation microenvironment. We hypothesize that, (1) nutritious litter (low C/N ratio) and suitable soil (high moisture, nutrient, and microbe) may facilitate litter decomposition; and (2) due to their distinct body sizes and diet preferences, cattle and sheep may pose different effects on soil property and litter quality that affect the litter decay rate.

With lower trampling, higher fecal nutrients, and less foraging on *L*. *chinensis*, sheep pose more positive effects on soil property and litter quality than cattle, thus increase litter decomposition.

## Results

### Litter decomposition

**Figure 1:**
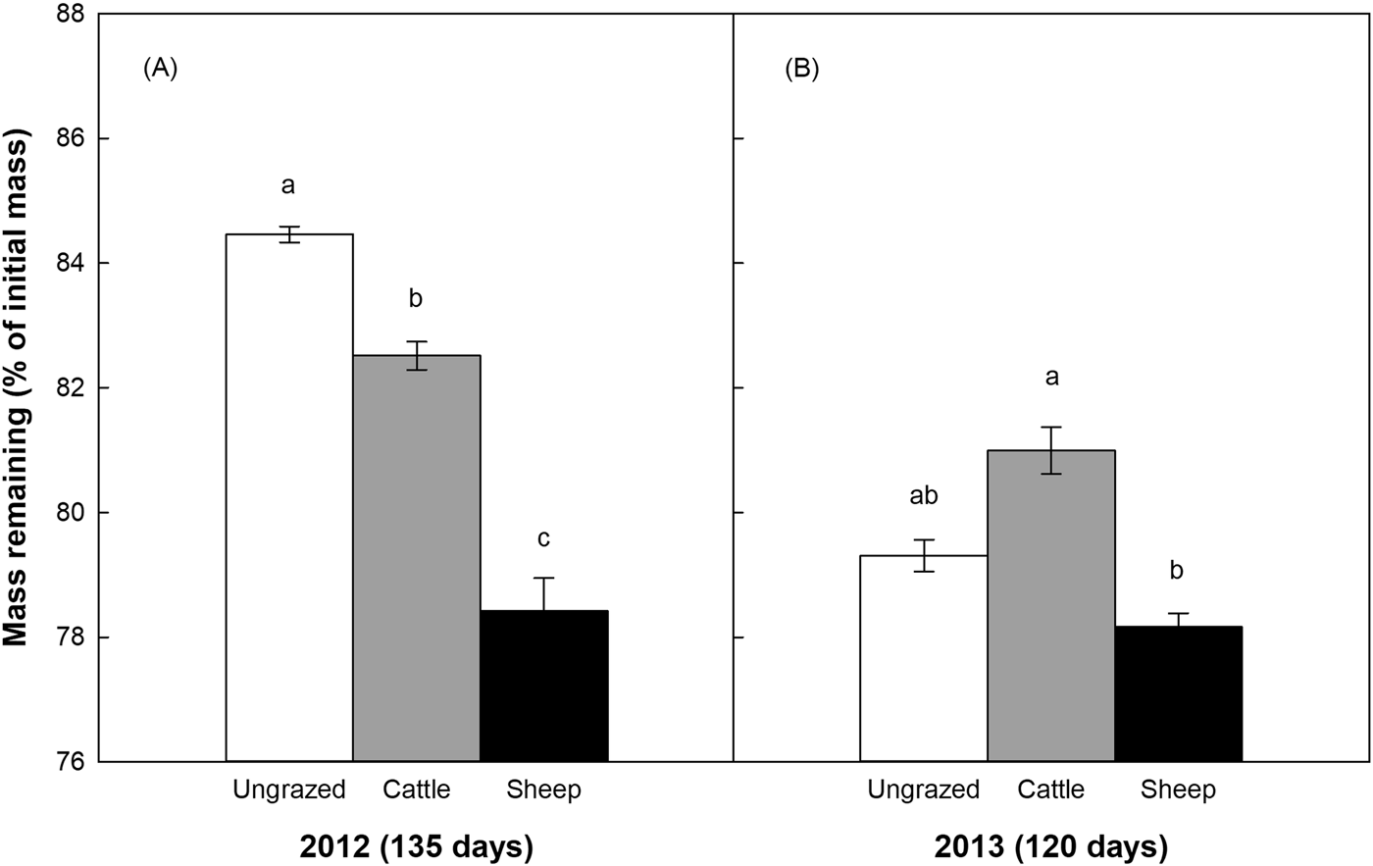
Litter mass remaining (mean ± SE) under the same initial quality but at different grazing sites after 135 days of incubation in 2012 (**A**), *n* = 4; and that under different initial qualities influence by different large herbivores at the same site after 120 days of decomposition in 2013 (**B**), *n* = 3. Different letters indicate significant differences between the treatments.

In our large-scale grazing experiment investigating the influences of changes in soil property on decomposition (experiment in 2012), litter mass remaining of *L*. *chinensis* grass ranged from 78% to 85% after 135 days of incubation. The mass loss of *L*. *chinensis* litter was significantly higher in sheep-grazed sites compared to other sites, and the ungrazed sites exhibited the lowest mass loss (Fig. 1A; Table 1). In the small-scale manipulative experiment investigating the role of plant litter quality (experiment in 2013), litter from the cattle-grazed sites had a significantly lower mass loss than that from the sheep-grazed sites, the amount of mass loss is reduced by nearly 3%, but litter mass loss did not differ between the ungrazed and grazed sites (Fig. 1B; Table 2).

**Table 1:** Litter mass remaining and general properties of surface soils of 0–10 cm in depth under different grazing treatments in 2012 (nested linear-mixed models analysis with grazing as the fixed factor, and plot as the random factor). *n* = 4.

Variables^†^	degree of freedom	*F*-value	*p*-value
MR	2, 45	27.529	<0.001
SM	2, 45	7.428	0.002
BD	2, 45	13.656	<0.001
pH	2, 45	8.435	0.001
EC	2, 45	5.219	0.009
TC	2, 45	0.779	0.465
TN	2, 45	6.386	0.004
C:N	2, 45	4.027	0.025
MBC	2, 45	2.068	0.138
NM	2, 45	3.642	0.034

^†^Key to abbreviations: MR = litter mass remaining; SM = soil moisture; BD = soil bulk density; pH = soil pH value; EC = soil electric conductivity; TC = soil total carbon; TN = soil total nitrogen; C:N = soil total C/N ratio; MBC = soil microbial biomass carbon; and NM = Net N mineralization rate.

**Table 2:** Litter mass remaining (*n* = 3) and main nutrients of *L*. *chinensis* standing litter (*n* = 4) under different grazing treatments in 2013 (nested linear-mixed models analysis with grazing as the fixed factor, and plot as the random factor).

Variables^†^	degree of freedom	*F*-value	*p*-value
MR	2, 42	4.040	0.025
LC	2, 33	2.116	0.137
LN	2, 33	105.149	<0.001
C:N	2, 33	117.541	<0.001

^†^Key to abbreviations: MR = litter mass remaining; LC = *L*. *chinensis* litter’s carbon content; LN = *L*. *chinensis* litter’s nitrogen content; and C:N = *L*. *chinensis* litter’s C/N ratio.

### Effects of herbivore grazing on soil properties and their relations to litter decomposition

**Figure 2:**
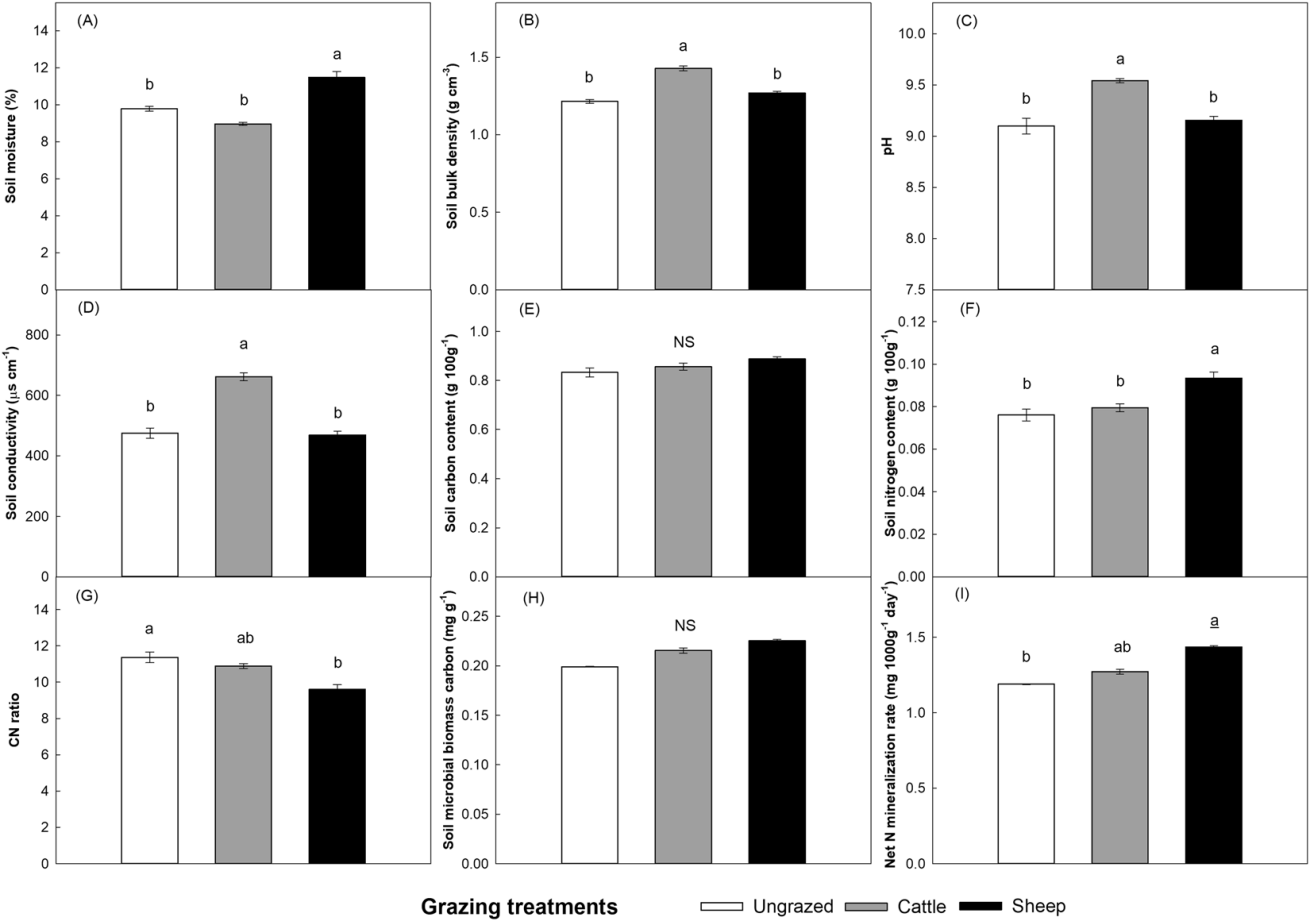
General properties (mean ± SE) of surface soils of 0–10 cm in depth under different grazing treatments in 2012. (**A**) moisture; (**B**) bulk density; (**C**) pH value; (**D**) electric conductivity; (**E**) total carbon content; (**F**) total nitrogen content; (**G**) total C/N ratio; (**H**) microbial biomass carbon; and (**I**) Net N mineralization rate. Different letters above the bars indicate significant differences. *n* = 4.

The sheep-grazed sites displayed a significantly higher soil moisture (Fig. 2A) compared to the cattle-grazed and the ungrazed sites, and the cattle-grazed sites had significantly higher soil bulk density (Fig. 2B), pH (Fig. 2C), and electric conductivity (Fig. 2D) compared to the sheep-grazed and the ungrazed sites. The sheep-grazed sites had a higher soil total nitrogen (Fig. 2F), but a lower total C/N ratio (Fig. 2G) compared to the ungrazed sites. The net N mineralization rate (NM) (Fig. 2I) in the sheep-grazed sites was significantly higher than in the ungrazed sites. Grazing did not have a significant impact on soil total carbon and microbial biomass carbon (MBC) (Fig. 2E,H; Table 1).

**Figure 3:**
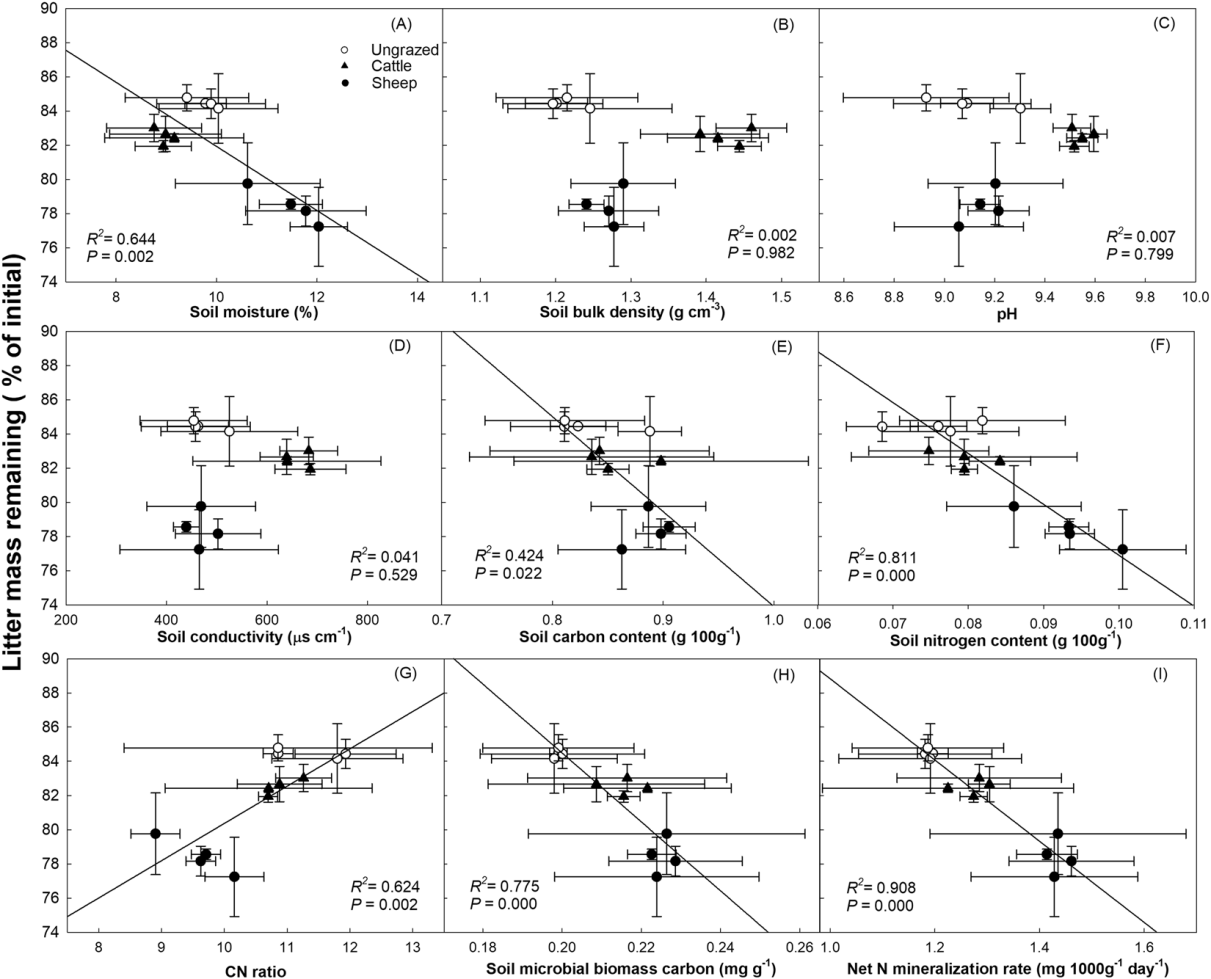
Relationships between litter mass remaining (on day 135 of incubation) and soil characteristics. (**A**) moisture; (**B**) bulk density; (**C**) pH value; (**D**) electric conductivity; (**E**) total carbon content; (**F**) total nitrogen content; (**G**) total C/N ratio; (**H**) microbial biomass carbon; and (**I**) Net N mineralization rate (mean ± SE). *n* = 12.

Under grazed conditions, litter mass remaining was negatively related to soil moisture (Fig. 3A), soil total carbon (Fig. 3E), soil nitrogen content (Fig. 3F), soil microbial biomass carbon (Fig. 3H), and soil net N mineralization rate (Fig. 3I). A positive linear relationship was found between litter mass remaining and soil C/N ratio (Fig. 3G).

### Effects of herbivore grazing on L. chinensis litter quality

**Figure 4:**
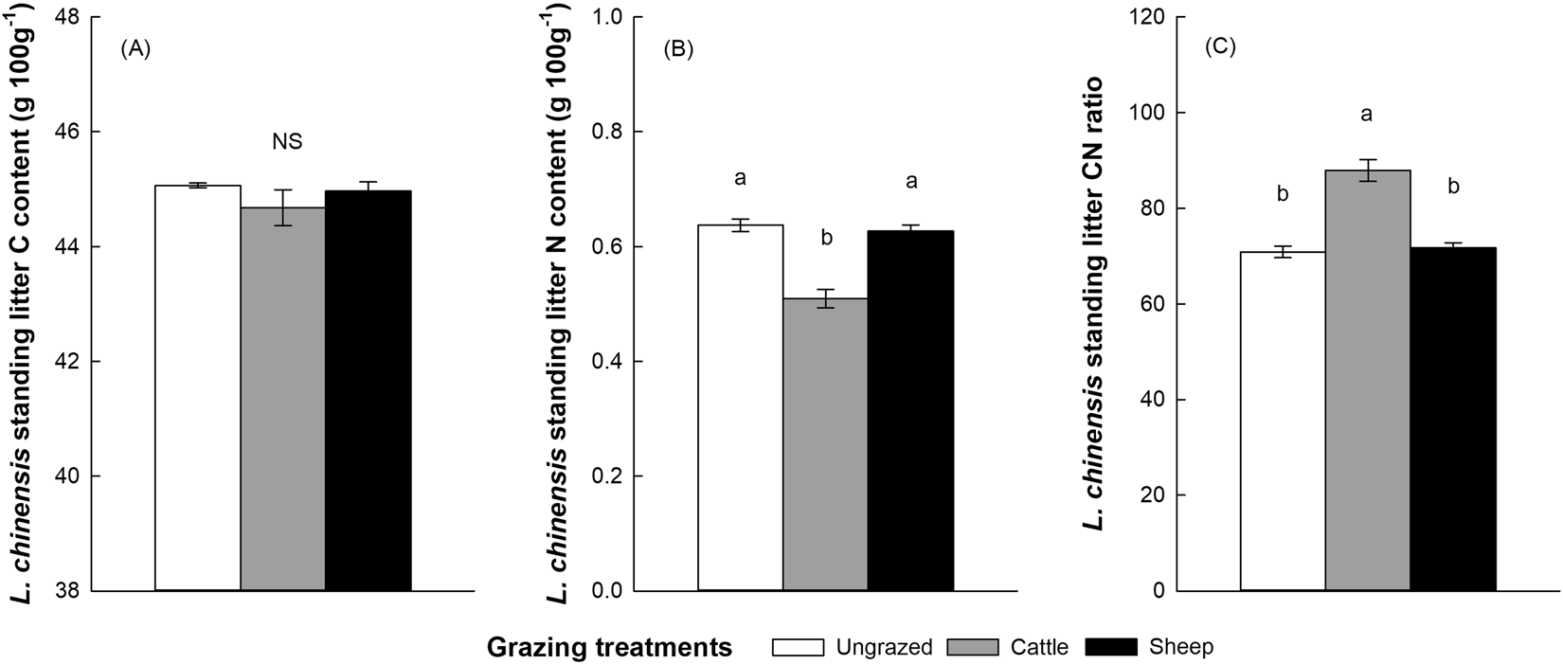
*Leymus chinensis* standing litter nitrogen content (**A**), carbon content (**B**), and C/N ratio (**C**) (mean ± SE) from different grazing treatments. Significant differences are indicated by different letters (NS = not significant). *n* = 4.

Cattle grazing considerably decreased *L*. *chinensis* litter’s nitrogen content (Fig. 4B) but increased soil C/N ratio (Fig. 4C) compared to the sheep-grazed and the ungrazed plots. There was no significant difference in the carbon content of *L*. *chinensis* litter among treatments (Fig. 4A; Table 2).

### Effects of herbivore grazing on plant density, coverage and height

**Figure 5:**
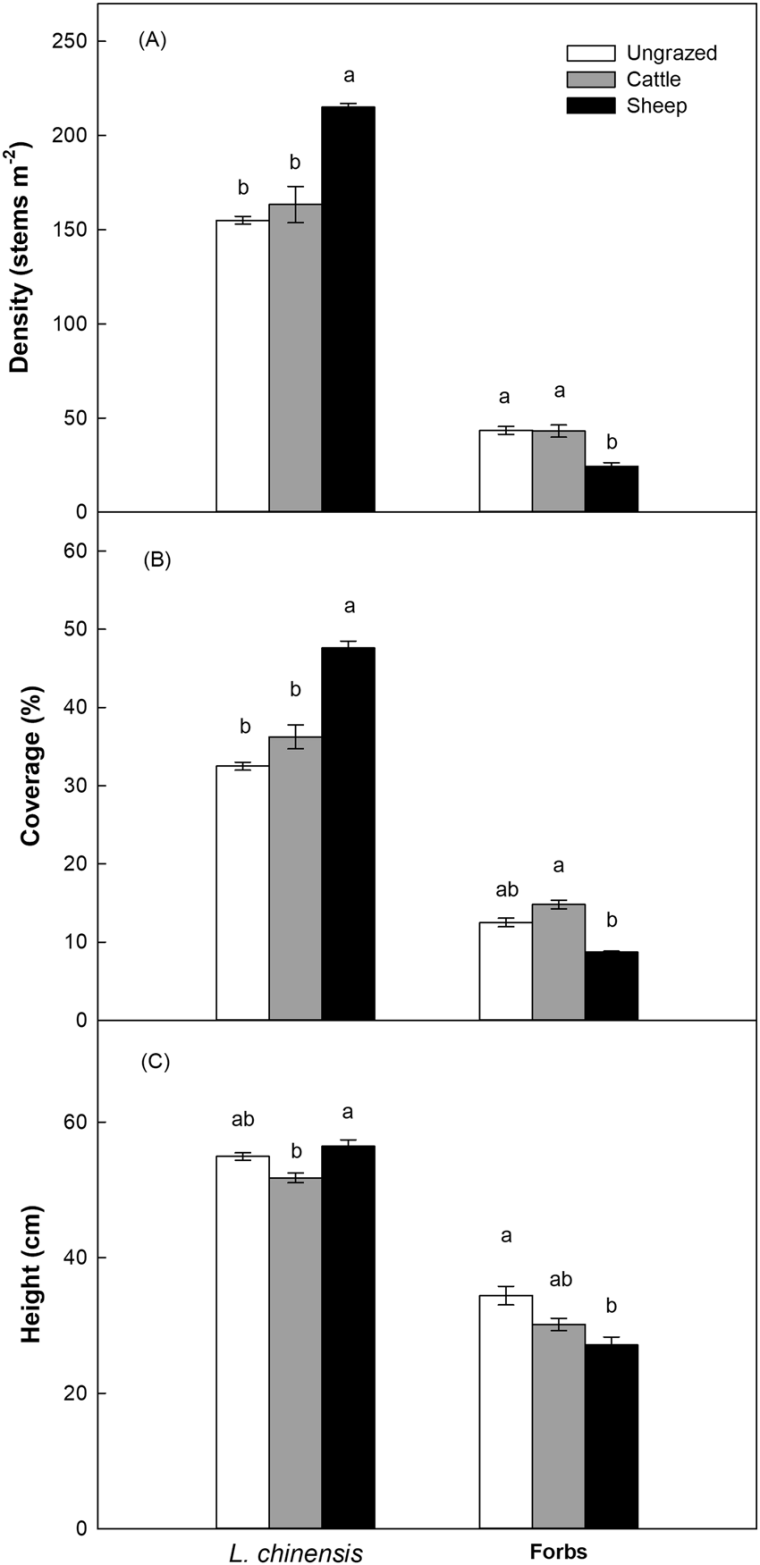
*Leymus chinensis* and forbs density (**A**), coverage (**B**), and height (**C**) were affected by different grazing treatments. Values represent mean ± SE. Different letters above the bars indicate significant differences. *n* = 4.

In general, *L*. *chinensis* had higher density, coverage, and height than forbs (Fig. 5). Sheep grazing significantly increased *L*. *chinensis* density but decreased forb density compared to cattle grazed and ungrazed plots (Fig. 5A). An increase in *L*. *chinensis* cover was found in sheep-grazed sites, but forb cover was lower in sheep-grazed sites than in cattle-grazed and ungrazed sites (Fig. 5B). Cattle grazing significantly reduced the height of *L*. *chinensis* compared to sheep grazing, while sheep grazing significantly decreased the height of forbs compared to the ungrazed plots (Fig. 5C; Table 3).

**Table 3:** *Leymus chinensis* and forbs density, coverage, and height under different grazing treatments in 2012 (nested linear-mixed models analysis with grazing as the fixed factor, and plot as the random factor). *n* = 4.

Variables^†^	degree of freedom	*F*-value	*p*-value
LD	2, 45	30.168	<0.001
LC	2, 45	28.480	<0.001
LH	2, 45	3.527	0.038
FD	2, 45	10.529	<0.001
FC	2, 45	6.287	0.004
FH	2, 45	3.465	0.040

^†^Key to abbreviations: LD = *L*. *chinensis* density; LC = *L*. *chinensis* coverage; LH = *L*. *chinensis* height; FD = forbs density; FC = forbs coverage; and FH = forbs height.

### Structural equation models (SEM)

**Figure 6:**
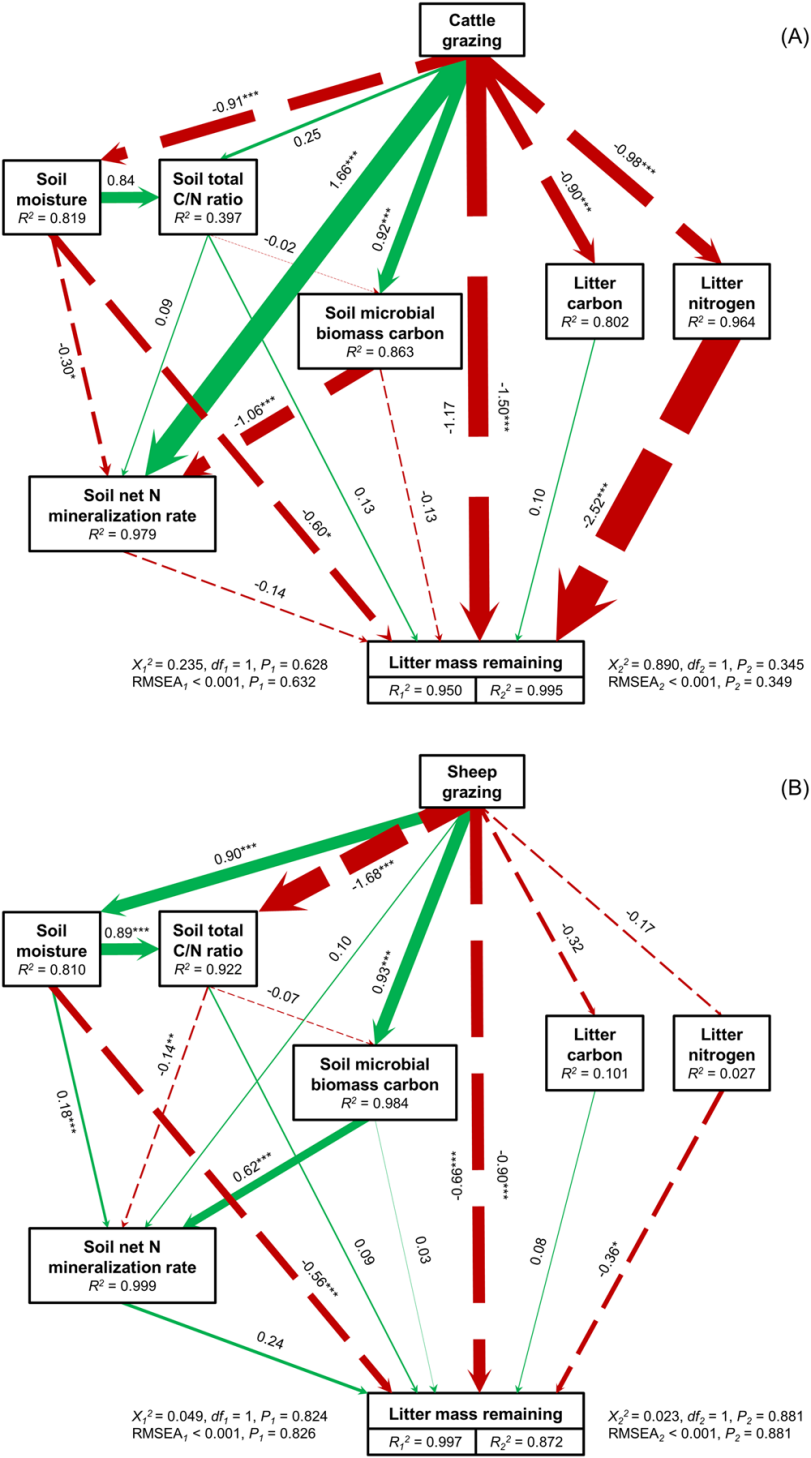
Structural equation models (SEM) depicting the control of cattle (**A**) vs. sheep grazing (**B**) on litter decomposition by affecting soil properties (*n* = 8) and litter quality (*n* = 6). The numbers on the arrows are standardized path coefficients, continuous and dashed arrows indicate positive and negative effects, respectively. The path widths are scaled proportionally to the path coefficient. The *R*^2^ value represents the proportion of total variance explained for the dependent variable of interest. Overall, goodness of-fit tests are shown in the bottom of the figure. *P < 0.05, **P < 0.01 and ***P < 0.001.

The fit between the final models and data were adequate for cattle grazing (${\chi }_{1}^{2}$ = 0.235, *P*_1_ = 0.628, RMSEA_1_ < 0.001, *P*_*1*_ = 0.632; ${\chi }_{2}^{2}$ = 0.890, *P*_*2*_ = 0.345, RMSEA_2_ < 0.001, *P*_*2*_ = 0.349), and sheep grazing (${\chi }_{1}^{2}$ = 0.049, *P*_1_ = 0.824, RMSEA_1_ < 0.001, *P*_*1*_ = 0.826; ${\chi }_{2}^{2}$ = 0.023, *P*_2_ = 0.881, RMSEA_2_ < 0.001, *P*_2_ = 0.881). Unstandardized and standardized path coefficients are summarized in Appendix C. The cattle models explained 95.0% of the variation in soil property and 99.5% of the variation in litter quality (Fig. 6A). The negative impact of cattle grazing on soil moisture reduced litter decomposition. In addition, cattle grazing decreased *L*. *chinensis* litter’s carbon and nitrogen content, and the negative effects on nitrogen content also reduce litter decomposition. The sheep models explained 99.7% of the variation in soil property and 87.2% of the variation in litter quality (Fig. 6B). The positive impact of sheep grazing on soil moisture accelerated litter decomposition. Unlike cattle, sheep grazing have no effect on *L*. *chinensis* litter’s carbon and nitrogen content, although there was still a significant correlation between nitrogen content and litter decomposition. Finally, grazing (cattle and sheep) had positive impacts on the MBC. Differently, cattle grazing accelerated NM, but sheep grazing reduced the soil total C/N ratio. Our SEM results show that grazing still has some unexplained impacts on decomposition.

## Discussion

Our study supports our hypothesis that cattle and sheep affect soil properties and litter quality, and thus litter decay rate, in different ways. That is, compared to sheep, cattle have more negative effects on soil properties such as bulk density, pH and conductivity (Fig. 2) and litter quality (C/N ratio) (Fig. 4) and thus to reduce litter decomposition. Specifically, our results provide experimental evidence that large herbivore grazing can affect litter decomposition of a dominant (host) grass, *L*. *chinensis*, by (1) altering the biotic and abiotic properties of soils, and by (2) altering the quality (C/N ratio) of plant litter. Under grazed conditions, these two pathways act to influence litter decomposition simultaneously (Fig. 6). In addition, litter decomposition depends on soil moisture and litter nitrogen content, and the effect of sheep grazing on the litter decomposition is more significant than cattle grazing (Figs 1 and 6). These findings help fill a critical information gap regarding whether and how grazing by large herbivores influences litter decomposition by altering soil conditions and litter chemistry in grasslands^38,46^.

### Large herbivore grazing affects litter decomposition by altering soil properties

In our study, grazing significantly altered the physicochemical properties of soils, particularly soil moisture, soil N content, as well as NM. These findings indicate that grazing-induced changes in soil properties could shape litter decomposition processes independent of changes in litter quality in the grazed areas. This conclusion is justified because we used plant litter with the same quality to test the influences of soil properties on decomposition in this experiment.

Our results are consistent with the findings of Cortez^47^ and Giese *et al*.^14^ who suggest that litter decomposition becomes more rapid with increases in soil moisture (Fig. 3A). Our results from SEM showed that, cattle pose a significant negative effect while sheep have a positive effect on soil moisture (Fig. 6). These contrasting results may be explained by the differences in body size of these two herbivores. The trampling activities of heavy cattle apparently decrease soil moisture by increasing the bulk density of soil (Fig. 2B), which makes soil surface water more difficult to permeate and easier to evaporate^48,49^. In addition to soil bulk density, soil evaporation could also be affected by vegetation cover, which may in turn influence soil water content^50,51^. And changes in plant community structure will affect the accumulation of soil surface litter, thus influencing the soil moisture^52,53^. In our experiment, sheep grazing significantly promotes the density and cover of *L*. *chinensis* (Fig. 5A,B), potentially enhancing soil moisture. Hence, herbivores can alter soil moisture by their indirect effects (trampling) on soil bulk density, and direct effects (herbivory) on vegetation abundance, which in turn affects litter decomposition in our systems.

Large herbivores have pronounced effects on nutrient conditions in grasslands via their influences on nutrient cycling and the inputs of nutrient-rich feces and urine into the soil^21^. The increase in soil nutrients can accelerate the litter decomposition by affecting soil microbial processes both directly through changing nutrient availability and indirectly by changing the inputs to microbial communities^6,54^. Our results show that both soil nutrients (total C and N content) and microbial communities (MBC and NM) are positively correlated with *L*. *chinensis* litter decomposition (Fig. 3E–I). Grazing increased soil total N content and NM (Fig. 2F,I), especially at the sheep-grazed sites where positive relationships between soil C/N ratio, microbial biomass carbon (MBC) and net N mineralization rate (NM) were found (Fig. 6B). Compared with cattle, sheep generally require more nutrients and select higher quality plants (higher N concentration), thus have feces with relatively higher nutrients^55,56^. This may partly explain why the soil N content at sheep-grazed sites was higher than at cattle-grazed sites (Fig. 2F).

We found that the soil N content was highly related to MBC and NM (Appendix A) thus may serve as a strong predictor of microbial abundance and activity that mediate litter decomposition rate. Soil nutrients therefore can indeed regulate the structure and function of microbial community^54,57^. Also, we found a significant positive interaction between soil C and N content that improves nutrient cycling (Appendix A).

Microbial communities largely determine the availability of C and N in soil. Previous research shows that herbivore foraging can stimulate microbial populations by changing plant carbon and nitrogen allocation to increase soil extractable carbon in the rhizosphere^27^. With high abundance and activity, microorganisms can efficiently use N, and thus accelerate N mineralization^58^. A lower C/N ratio in the microbial substrate also has been related to a higher N mineralization^6^. In our study, we found a close relationship between MBC and NM (Appendix A). Herbivores may also increase the growth of microbes and nutrient cycling by returning feces^21^ and influence the microbial abundance by trampling^59^. This may partly explain why herbivore grazing substantially increases soil microbial abundance and activity, and eventually and positively impacts litter decomposition.

### Large herbivore grazing affects litter decomposition by altering litter quality

Selective grazing affects nutrient transportation and allocation, thereby changing chemical composition of the plant material^60^. Plant regrowth following defoliation is often considered to increase nutrient concentrations in tissues, which enhances the subsequent decomposition rates^13,40^. Contrary to these observations, there is also evidence that long-term grazing can induce a tolerant response in plants. For instance, grazing can make plants allocate more C but less N and lower the nutrient value (higher C/N ratio) in plant tissue, thereby avoid consumption by herbivores. Such changes in plant nutrients may result in low-quality litter inputs into the soil, and therefore reduce decomposition rate^44,61^.

Our results support the previous findings that grazing can cause changes in plant litter quality^11,12^. In our study, the litter samples from cattle-grazed sites have lower N concentration and higher C/N ratio than those from sheep-grazed and ungrazed sites (Fig. 4). There is a significant difference in the disappearance of litter mass among treatments (Fig. 1B), implying a causal relationship between plant litter quality (C/N ratio) and litter decomposition. In our system, cattle are generalist while sheep are specialist herbivores, cattle mainly feed on the dominant *L*. *chinensis*, and rarely feed on other plant species. In contrast, sheep mainly feed on the N-rich forbs, whereas rarely feed on the N-poor *L*. *chinensis* grass (Fig. 5)^62^. After a long-term (5-year) grazing, *L*. *chinensis* may have already adapted the pressure from cattle grazing by allocating more N into belowground components, especially roots, and by allocating less N into leaves and shoots to avoid further herbivory, a strategy for herbivory defense. In contrast, litter quality (C/N ratio) of *L*. *chinensis* from sheep-grazing does not change compared to the ungrazed sites, simply because sheep do not directly feed on this grass. This also indicates that sheep grazing may be less likely to affect *L*. *chinensis* litter decomposition by changing the quality of plant litter, comparing to the strong effect of cattle on litter decomposition via this pathway (Fig. 6).

## Conclusion

Taken together, our study shows that large herbivores can influence plant litter decomposition independently through altering both soil properties (especially soil moisture) and the quality (especially litter nitrogen content) of plant litter itself. Herbivore grazing can influence litter decay rate by changing soil moisture, soil N content, soil microbial biomass C, N mineralization, as well as via increasing or decreasing plant tissue C/N ratio. These two pathways may occur simultaneously, and may well interact with each other to control litter decomposition processes in grazing systems. Our study also suggests that different large herbivores (cattle vs. sheep) may exert distinctive or even opposite effects on litter decomposition, mainly due to their different influences on the properties and the quality of plant litter^40,54^. Compared to the impact of cattle on both pathways (Fig. 6A), sheep control litter decomposition only by affecting biotic and abiotic soil characteristics (Fig. 6B). Hence, an improved understanding of how herbivores grazing can affect litter decomposition will help us to better predict the changes in nutrient dynamics in grazing ecosystems.

## Methods

### Study site

The study site was situated in the Grassland Ecological Research Station of Northeast Normal University, Jilin Province, P. R. China (44°45′ N, 123°45′ E). This site has a semi-arid continental monsoon climate with subdued topography. Annual mean temperature ranges from 4.6 to 6.4 °C and annual precipitation is 280 to 400 mm with about 90% falling during the growing season (from May to October), and the frost-free period is about 140 days. This region is dominated by the perennial grass *L*. *chinensis*, which accounted for 50–70% of the total aboveground biomass^63^. *Leymus chinensis* is a widespread dominant grass, it typically dominates arid and semi-arid steppe in northern China, eastern Mongolia and Transbaikalia, Russia^64^. Following senescence, a very large proportion of the standing dead materials usually remains attached to *L*. *chinensis*. There are many companion species including grasses: *Phragmites australis* Trin. ex Steud., *Calamagrostis epigejos* Roth and *Chloris virgata* Swartz; and forbs: *Kalimeris integrifllia* Turcz. ex DC., *Artemisia scoparia* Waldst. et Kit., *Artemisia anethifolia* Web. ex Stechm., *Potentilla flagellaris* Willd. ex Schlecht. and *Carex duriuscula* C. A. Mey.^65^. The main soil type of this habitat is a meadow soil with high salinity and alkalization (pH 8.3–10.0). The soils in the area are relatively homogeneous and deficient in many important nutrients, particularly phosphorus, magnesium, and calcium^62^.

The northeast fine-wool sheep and Simmental cattle are the most important vertebrate herbivores^55^, whose density or grazing pressure is controlled by human agrarian practices. Cattle are generalist herbivores; they mainly feed on the dominant plant species, *L*. *chinensis* grasses. In contrast, sheep are specialist herbivores; they mainly feed on forb species, while rarely feeding on the *Leymus* grasses^60^. Our experiments were conducted within twelve 25 m × 25 m plots, which were randomly assigned one of three treatments: sheep-grazed, cattle-grazed, or ungrazed. These treatments each had four replicates. All plots were established and grazing was performed within a fenced experimental site since 2008. The distances between plots ranged from 50 to 250 m. Each plot consisted of four 3 m × 3 m subplots. From 2008 to 2012, grazing activity occurred from early June to early September each year, with a moderate stocking rate of 0.3–0.5 animal units/ha in these plots^66^.

All experimental protocols, including animal ethics, were approved by the Jilin Academy of Agricultural Science (Changchun city, Jilin Province, China), and all methods were performed in accordance with Regulation on the Administration of Laboratory Animals (2017 Revision, State Council, Beijing, China).

### Impacts of grazing-induced changes in soil condition on litter decomposition

#### Litter sampling

The aboveground standing litter was harvested from one pure *L*. *chinensis* community at the ungrazed plots in early October 2011. Each selected individual plant was approximately 16–17 cm long, with 4 leaves attached to the stem so that the litter quality would be roughly homogeneous. After cleaning with water, the selected materials were oven-dried at 80 °C for 48 hours or until they reached constant mass.

#### Litter decomposition

Litter decomposition was studied using the litterbag method. We filled the nylon mesh bags (10 cm × 10 cm, 2 mm pore) with approximately 10 g dry litter (cut into about 8 cm long pieces). The mesh allows retention of litter during fragmentation, thus preventing the loss of litter from the bags. The decomposers were allowed to enter the bags through the mesh so that litter residues instead of mere litter decomposition could be measured. On June 5, 2012, a total of 192 litterbags were deployed in all 48 subplots. Within each subplot, we randomly assigned four 0.5 m × 0.5 m incubation quadrats, each with one litterbag in it. Every subplot was randomly positioned beneath a pure *L*. *chinensis* community. All litterbags were fixed by nails (7 cm in depth) in the top humus layer and covered with humus and surface litter found in place to maintain constant environmental conditions^67^ and to reduce the chance of being explored by large herbivores.

Litterbags were collected on October 20, 2012, 135 days after litter exposure. Each retrieved litterbag was carefully emptied and washed clear of external soil, and every four litterbags within each subplot were composited into one. A total of 48 contents were oven-dried at 80 °C for 48 hours and weighed. Mass remaining was calculated as the difference between the initial dry mass and the actual dry mass of litter in the bags.

#### Soil sampling and measurements

In August 2012, after removing the litter layer, soils were sampled from the top 10 cm from three randomly distributed locations within each litterbag incubation quadrat (0.5 m × 0.5 m) using a soil corer (2.5-cm in diameter). Three soil cores were collected and then mixed into one within each quadrat, and then, all these four mixed soils were pooled into one single sample in each subplot. A total of 48 fresh soil samples were collected from all 48 subplots and kept cold in the field until laboratory preparation. In the laboratory, the samples were sieved with a 2-mm mesh, and each sample was separated into two sub-samples, one for measuring soil physico-chemical properties after being air-dried, and the other was stored at 4 °C for measuring microbial biomass carbon. The soils for measuring bulk density were collected next to the sampled soils from the same layer using a cutting ring with the volume of 100 cm^3^ within each quadrat, and then pooled into one single sample for each subplot.

Soil net N mineralization rates were measured *in situ* within all 48 subplots from August 1 to 31, 2012 using the method of Hazelden and Boorman^68^. A pair of soil cores were collected in each subplot from the upper 10 cm of soil (the litter layer was carefully removed) using PVC tubes of 2.5 cm in diameter. One core was kept cool and brought to the laboratory, and the other was re-buried in the soil and incubated *in situ* for 30 days. The top and bottom of the tubes were sealed with plastic lids, and there were two gas-permeable holes below the top lid and above the soil^69^. After the incubation period, the tubes were kept cool during transportation to the laboratory, and the content of each tube was homogenized and sieved with a 2-mm mesh.

We used the gravimetric method to determine soil moisture by weighing soil after drying at 105 °C for 12 hours. We used W/V (W is the soil weight in grams after drying at 105 °C for 12 hours, and V is 100 cm^3^) to estimate soil bulk density (g cm^−3^)^70^. We used sediment slurries (10 g air-dry soil and 50 ml deionized water shaken for 1 hour, 250 rpm) to measure soil pH and electric conductivity^71^. Soil total carbon and nitrogen contents were determined by an automated element analyzer (Vario EL cube, Elementar Analysensysteme GmbH, Hanau, Germany) after the air-dried soil samples were sieved by a 0.15 mm mesh.

We used the chloroform fumigation-extraction (CFE) technique^72^ to estimate microbial biomass. Soil samples (10 g) were extracted with 0.5 M K_2_SO_4_ in a ratio 1:5, and soil extracts were shaken in an orbital shaker at 300 rpm for 30 minutes at 20 °C and filtered through Whatman no. 1 filter paper^73^. The filtered extracts (CHCl_3_^-^ fumigated and unfumigated) were obtained to measure microbial biomass carbon (MBC) using a TOC analyzer (vario TOC select, Elementar Analysensysteme GmbH, Hanau, Germany). Soil MBC (mg g^−1^) was calculated as the difference between the fumigated sample and its paired unfumigated sample. We used a continuous flow analyzer (Futura II, Alliance Instruments Ltd., France) to determine soil available N content (NH_4_^+^ and NO_3_^−^), and 10 g of fresh soil (initial and the incubated) was extracted with 50 ml of 1 M KCl and filtered to pass a 0.45-μm Millipore filter. The daily net mineralization rates were considered to be the difference in available N between the initial and incubated samples divided by the number of days of incubation (mg kg^−1^ day^−1^).

#### Plant measurements

In August 2012, we determined the proportion of *L*. *chinensis* and forbs by counting the number of individuals for each species (counting the ramet number of individuals for clonal species) and recorded species composition within each litterbag incubation quadrat (0.5 m × 0.5 m). The percent live plant canopy cover and height of live foliage were measured at the same time. The percentage cover was estimated visually in each quadrat (with a maximum of 100% total cover). Plant height was measured on five randomly selected individual plants of both *L*. *chinensis* and forbs in quadrats.

### Impacts of grazing-induced changes in litter quality on litter decomposition

#### Litter chemistry analyses

At the end of October 2012 when all aboveground plants were dead, we collected *L*. *chinensis* standing litter by clipping at 2-cm above the soil surface in the ungrazed, sheep-grazed, and cattle-grazed plots. The litter materials were cleaned with water and oven-dried at 80 °C for 48 hours. Then, a ball mill (MM 400, Retsch GmbH, Hann, Germany) was used to grind the mixture of leaves and stems into fine powder. We measured the total carbon and nitrogen contents in pulverized litter materials from the litter mixture (leaves and stems) by an automated element analyzer (Vario EL cube, Elementar Analysensysteme GmbH, Hanau, Germany).

#### Litter decomposition

Given that herbivore grazing may significantly alter the litter quality (C/N ratio) of *L*. *chinenesis* (Fig. 4), we further conducted another decomposition experiment to independently investigate how grazing-induced changes in plant litter chemistry may influence decomposition processes. We established three 5 m × 5 m plots with five replicated incubation quadrats (0.5 m × 0.5 m) in each plot in a protected natural field of the nearby grazing regions. The distances between plots ranged from 3 to 5 m. The litter materials of *L*. *chinensis* collected from 3 of 4 plots under different grazing treatments were separately filled into litterbags (10 cm × 10 cm). Litter in each bag was cut into 8-cm long pieces to simulate physical breakdown by large herbivore trampling and comminution by soil fauna^40^. Litterbags were made of 2 mm mesh nylon fabric and filled with 5 g of dry litter.

On June 15, 2013, a total of 45 litterbags were deployed in all 15 incubation quadrats. Each quadrat had three bags filled with litter from sheep-grazed, cattle-grazed, and ungrazed treatments, respectively. On October 15, 2013 (120 days after incubation), litterbags were collected and the contents were cleaned, dried and weighed. Mass remaining was calculated as the difference between the initial dry mass and the actual dry mass of litter in the bags at the sampling date.

#### Statistical analysis

We used linear-mixed models (LMMs) to analyze the effects of large herbivore grazing on soil properties (moisture, bulk density, pH value, electric conductivity, C and N content, microbial biomass carbon, and net N mineralization rate), plant community composition (relative density, coverage and height), litter quality (C and N content), and the changes in the litter decomposition (litter mass remaining, 2012 and 2013), with grazing as the fixed factor and plot as the random factor. Tukey’s multiple comparison was used to evaluate the differences among the grazing treatments. Linear regression analysis was conducted to examine the relationship between litter mass and soil properties. Pearson correlation analysis was conducted to examine the covariation between selected variables (e.g., soil properties) and litter decomposition.

We used the structure equation model (SEM) to assess the direct and total effects of grazing-induced changes in soil microenvironment and litter quality potentially affecting litter decomposition. We created two models, i.e., ‘cattle grazing’ and ‘sheep grazing’, based on prior and theoretical knowledge (Appendix B). The paths selection and adjustment were based on our data analysis results of linear regression and Pearson correlation. The adequacy of the model fitting was assessed in several ways, including the Chi-square test (*χ*^*2*^-test) and the root square mean error of approximation (RMSEA). Favorable model fits were suggested by no significant difference on the *χ*^*2*^-test (*P* > 0.05) and low RMSEA (values < 0.05 and *P* ≤ 1.00)^74^. To improve the model fit, we then added three paths from soil moisture to soil total C/N ratio, from soil total C/N ratio to litter mass remaining and from grazing to litter mass remaining, and removed the path from soil moisture to microbial biomass carbon (Fig. 6).

All of the statistical tests were performed with R 3.4.3 (http://cran.r-project.org). LMMs was performed using the *lmer* function form the package ‘lme4′ in R. SEM was performed using the ‘lavaan’ package.

## Electronic supplementary material

Dataset 1
