# Supplementary material for: Large herbivores influence plant litter decomposition by altering soil properties and plant quality in a meadow steppe
Source: Sci Rep. 2018 Jun 14;8:9089. doi: 10.1038/s41598-018-26835-1 (PMC6002471; doi:10.1038/s41598-018-26835-1)
Supplement: Supplementary file 1 — Dataset 1 [file 41598_2018_26835_MOESM1_ESM.zip › Appendix.pdf]

Large herbivores influence plant litter decomposition by altering soil properties and plant quality in a meadow steppe

Zhongnan Wang[1], Xia Yuan[1], Deli Wang[1*], Yang Zhang[1], Zhiwei Zhong[1], Qinfeng Guo[2], Chao Feng[1]

[1] Key Laboratory of Vegetation Ecology, Ministry of Education; Institute of Grassland Science/School of Environment, Northeast Normal University, and Changchun, Jilin 130024, China

[2]USDA FS, Eastern Forest Environmental Threat Assessment Center, RTP, NC 27709, USA

*Corresponding author: Deli Wang

Tel: 86-431-85099737; Fax: 86-431-85695065

Email: wangd@nenu.edu.cn

Supporting Information

Additional supporting information may be found in the online version of this article:

Appendix A. Pearson correlations between litter decomposition (mass remaining, year 2012) and soil variables.

Variables†	MR	SM	BD	pH	EC	TC	TN	C:N	MBC	NM
MR	1	-0.803	-0.044	0.082	0.202	-0.651	-0.900	0.790	-0.880	-0.953
SM	**0.002**	1	-0.534	-0.563	-0.689	0.505	0.792	-0.586	0.500	0.730
BD	0.892	0.074	1	0.898	0.950	0.139	-0.081	-0.016	0.381	0.068
pH	0.799	0.057	**0.000**	1	0.931	0.228	-0.226	0.143	0.222	-0.044
EC	0.529	**0.013**	**0.000**	**0.000**	1	0.000	-0.307	0.272	0.121	-0.179
TC	**0.022**	0.094	0.667	0.476	0.999	1	0.618	-0.562	0.698	0.593
TN	**0.000**	**0.002**	0.803	0.480	0.332	**0.032**	1	-0.748	0.732	0.807
C:N	**0.002**	**0.045**	0.961	0.658	0.392	0.057	**0.005**	1	-0.796	-0.855
MBC	**0.000**	0.098	0.222	0.487	0.707	**0.012**	**0.007**	**0.002**	1	0.871
NM	**0.000**	**0.007**	0.834	0.893	0.577	**0.042**	**0.002**	**0.000**	**0.000**	1

† Key to abbreviations: MR = litter mass remaining; SM = soil moisture; BD = soil bulk density; pH = soil pH value; EC = soil electric conductivity; TC = soil total carbon; TN = soil total nitrogen; C:N = soil total C/N ratio; MBC = soil microbial biomass carbon; and NM = Net

N mineralization rate. The values on the lower-left side of the diagonal are *P* values and the bold values represent significant correlations.

Appendix B. Structural Equation Model (SEM) procedures and the priori SEM model.

In the present study, model fitting was evaluated using a maximum likelihood Chi-square goodness-of-fit test (χ^2-test) and the root square mean error of approximation (RMSEA). Under the χ^2 test, a good model should have a P value > 0.05, thus a better model fit has a larger P value. The RMSEA avoids issues of sample size by analyzing the discrepancy between the hypothesized model, with optimally chosen parameter estimates, and the population covariance matrix. The RMSEA ranges from 0 to 1, with smaller values indicating better model fit, and typically does not exceed 0.08. Value < 0.05 is indicative of good model fit.

We used theoretical knowledge to develop a priori model (Fig. S1) to display the predicted causal relationships among the variables operating in our system. Our priori model depicted that from grazing (cattle or sheep) to litter mass remaining (MR), there would be a number of indirect effects, mediated by soil moisture (SM), soil C/N ratio (C/N), soil microbial biomass carbon (MBC), soil net N mineralization rate (NM) and litter quality (C and N content), separately. The arrows (pathways) represent hypothesized causal relationships anticipated to exist between variables. That is, under the influence of grazing (foraging, trampling and feces), MR may be affected by SM, MBC, NM and litter quality. In addition, SM and C/N can alter MR

by affecting MBC and NM. And MBC acts as an intermediate variable, to change MR by influencing NM (Fig. S1).

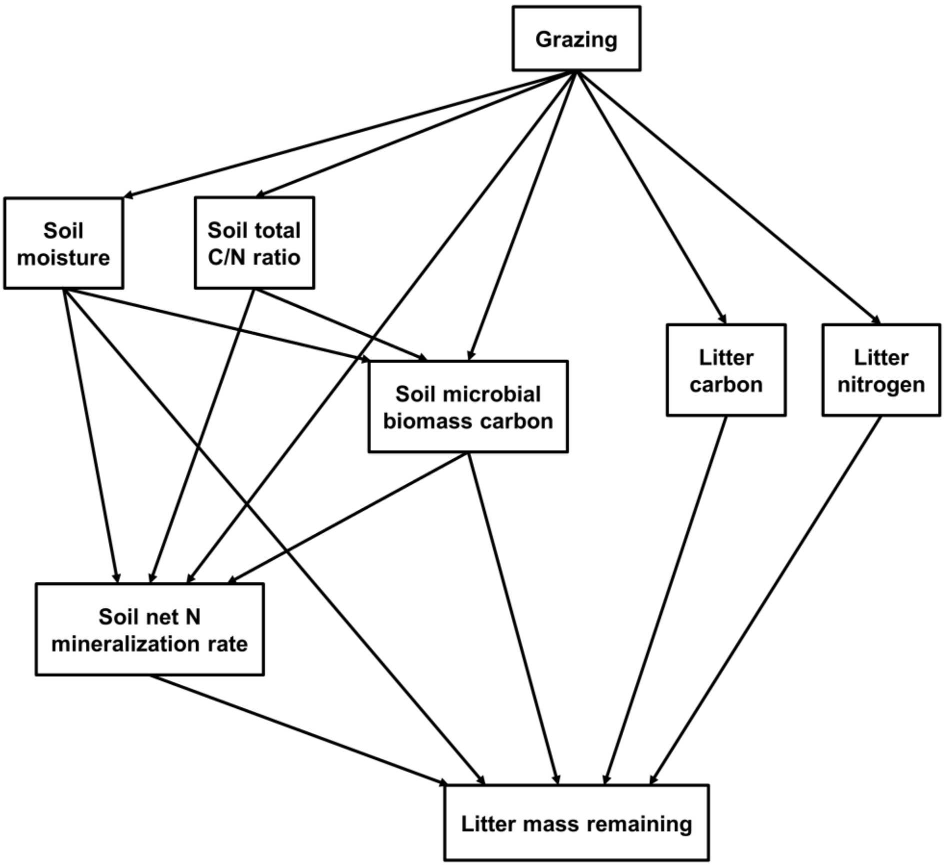

Figure S1. A priori model showing the predicted relationships among grazing, soil moisture, soil C/N ratio, soil microbial biomass carbon, soil net N mineralization rate and litter quality.

References

Eldridge, D. J., Delgado-Baquerizo, M., Travers, S. K., Val, J. & Oliver, I. Do grazing intensity and herbivore type affect soil health? Insights from a semi-arid productivity gradient. *Journal of Applied Ecology* **54**, 976-985 (2017).

Appendix C. Full model results including unstandardized and standardized path coefficients in Fig. 6, with unstandardized path coefficients (Unstandard. coefficients), standard error of the regression weight (S.E.), critical value for the regression weight (*z*-value), level of significance for the regression weight (*P*-value) and standardized path coefficients (Standard. coefficients). For more information on the model fit, see the primary text. The arrows represent causal relations, with the variables in the first column being affected by the variables in the second column.

Table C1. The path analysis revealing the effects of cattle grazing on litter decomposition by influencing soil properties.

		Unstandard. coefficients	S.E.	*z*-value	*P* value	Standard. coefficients
Litter mass remaining	← Cattle grazing	-0.012	0.010	-1.257	0.209	-1.174
Litter mass remaining	← Soil moisture	-0.135	0.059	-2.287	0.022	-0.599
Litter mass remaining	← Soil total C/N ratio	0.290	0.253	1.147	0.251	0.131
Litter mass remaining	← Soil microbial biomass carbon	-0.154	0.704	-0.219	0.827	-0.133
Litter mass remaining	← Soil net N mineralization rate	-0.031	0.119	-0.261	0.794	-0.141
Soil moisture	← Cattle grazing	-0.041	0.007	-6.010	0.000	-0.905
Soil total C/N ratio	← Cattle grazing	0.001	0.003	0.382	0.702	0.247
Soil total C/N ratio	← Soil moisture	0.086	0.065	1.309	0.190	0.844
Soil microbial biomass carbon	← Cattle grazing	0.008	0.001	5.992	0.000	0.916

Soil microbial biomass carbon	← Soil total C/N ratio	-0.046	0.295	-0.158	0.875	-0.024
Soil net N mineralization rate	← Cattle grazing	0.077	0.008	9.340	0.000	1.658
Soil net N mineralization rate	← Soil moisture	-0.309	0.137	-2.261	0.024	-0.303
Soil net N mineralization rate	← Soil total C/N ratio	0.944	0.671	1.408	0.159	0.094
Soil net N mineralization rate	← Soil microbial biomass carbon	-5.521	0.729	-7.572	0.000	-1.060

Table C2. The path analysis revealing the effects of cattle grazing on litter decomposition by influencing litter quality.

		Unstandard. coefficients	S.E.	*z*-value	*P* value	Standard. coefficients
Litter mass remaining	← Cattle grazing	-0.029	0.003	-9.669	0.000	-1.503
Litter mass remaining	← Litter carbon content	0.703	0.427	1.667	0.096	0.103
Litter mass remaining	← Litter nitrogen content	-0.393	0.023	-17.329	0.000	-2.516
Litter carbon content	← Cattle grazing	-0.003	0.001	-4.924	0.000	-0.895
Litter nitrogen content	← Cattle grazing	-0.121	0.010	-12.626	0.000	-0.982

Table C3. The path analysis revealing the effects of sheep grazing on litter decomposition by influencing soil properties.

			Unstandard. coefficients	S.E.	*z*-value	*P* value	Standard. coefficients
Litter mass remaining	←	Cattle grazing	-0.041	0.011	-3.615	0.000	-0.664
Litter mass remaining	←	Soil moisture	-0.183	0.038	-4.854	0.000	-0.560
Litter mass remaining	←	Soil total C/N ratio	0.289	0.297	0.973	0.331	0.094
Litter mass remaining	←	Soil microbial biomass carbon	0.077	0.791	0.098	0.922	0.033
Litter mass remaining	←	Soil net N mineralization rate	0.061	0.125	0.484	0.628	0.242
Soil moisture	←	Cattle grazing	0.170	0.029	5.836	0.000	0.900
Soil total C/N ratio	←	Cattle grazing	-0.034	0.005	-7.425	0.000	-1.679
Soil total C/N ratio	←	Soil moisture	0.095	0.024	3.935	0.000	0.890
Soil microbial biomass carbon	←	Cattle grazing	0.025	0.002	10.086	0.000	0.932
Soil microbial biomass carbon	←	Soil total C/N ratio	-0.090	0.122	-0.737	0.461	-0.068
Soil net N mineralization rate	←	Cattle grazing	0.024	0.031	0.770	0.441	0.096
Soil net N mineralization rate	←	Soil moisture	0.238	0.065	3.645	0.000	0.182
Soil net N mineralization rate	←	Soil total C/N ratio	-1.746	0.567	-3.078	0.002	-0.142
Soil net N mineralization rate	←	Soil microbial biomass carbon	5.727	0.947	6.046	0.000	0.615

Table C4. The path analysis revealing the effects of sheep grazing on litter decomposition by influencing litter quality

		Unstandard. coefficients	S.E.	*z*-value	*P* value	Standard. coefficients
Litter mass remaining	← Cattle grazing	-0.012	0.002	-5.736	0.000	-0.895
Litter mass remaining	← Litter carbon content	0.621	1.173	0.530	0.596	0.082
Litter mass remaining	← Litter nitrogen content	-0.321	0.134	-2.400	0.016	-0.355
Litter carbon content	← Cattle grazing	-0.001	0.001	-0.823	0.411	-0.318
Litter nitrogen content	← Cattle grazing	-0.002	0.006	-0.410	0.682	-0.165
